# Epidermal nevus syndrome with the mutation of *PTCH1* gene and cerebral infarction: a case report and review of the literature

**DOI:** 10.1186/s13256-022-03547-9

**Published:** 2022-09-28

**Authors:** QingQing Deng, Yan Li, ZhanLi Liu, JieLin Zhou, LingWei Weng

**Affiliations:** Department of Pediatrics, Hangzhou Children’s Hospital, 195 Wenhui Road, Hangzhou, 310014 Zhejiang People’s Republic of China

**Keywords:** Epidermal nevus syndrome, *PTCH1* gene, Cerebral infarction, Paralysis, Cerebrovascular malformation

## Abstract

**Background:**

Epidermal nevus syndrome is a group of congenital neuroectodermal and/or mesodermal disorders characterized by the epidermal nevi in common association with cerebral, eye, skeletal, cardiovascular, and renal abnormalities. Epidermal nevus syndrome is a rare syndrome, and epidermal nevus syndrome with the mutation of *PTCH1* gene and cerebral infarction is even rarer and has not been reported to the best of our knowledge.

**Case presentation:**

We report the case of a 10-month-old Chinese female patient who presented to our pediatric neurologic department, University of Wenzhou medical teaching Hospital, Hangzhou. She has mobility disorders on the right limbs and recurrent seizures. She had congenital disorder accompanied by brownish-black and verrucose plaques on the right side of the face as well as extensive brownish-black plaques and brown nevi on the right side of the trunk and the right arm. Epidermal nevus syndrome was diagnosed on the basis of her symptoms. Somatic sebaceous nevi and hypoplastic defects of skin, cerebra, eyes, skeleton, and cardiovascular and renal system were observed. However, in addition to the typical clinical characteristics, the patient also has a mutation (c.109G > T) in *PTCH1* gene and cerebral infarction. We present a novel case report and literature review.

**Conclusion:**

To our knowledge, epidermal nevus syndrome with a mutation of *PTCH1* gene and cerebral infarction has not been reported previously. This case report may contribute to characterizing the phenotype of epidermal nevus syndrome, help clinicians be aware of the association of this condition with *PTCH1* gene and cerebral infarction, raise clinical suspicion, and improve early therapy.

## Background

Epidermal nevus syndrome (ENS) is a group of congenital neuroectodermal and/or mesodermal disorders characterized by epidermal nevi in association with cerebral, eye, skeletal, cardiovascular, and renal abnormalities [1] that can affect a range of organs. The occurrence of epidermal nevus syndrome is very rare. Solomon *et al*. reported the first case of epidermal nevus syndrome in 1968 [2]. While diagnostic criteria have not been defined yet, the diagnosis of ENS is mostly clinical. The universal diagnosis is usually based on sebaceous nevus of the scalp, truncus, or extremities present at the time of birth or in early childhood, accompanied by various anomalies of the central nervous system, skeletal system, kidneys, and eyes [3], although central nervous system involvement has been noted to be the most common systemic association with ENS, presenting with a constellation of signs and symptoms ranging from refractory epilepsy to focal motor deficits and developmental delay [4]. Cerebral infarction associated with epidermal nevus syndrome is rare, and the molecular mechanisms of epidermal nevus syndrome spectrum are unclear. We further reviewed the current published case report of ESN and discussed the molecular mechanisms. Recent reports of somatic mosaicism in the pathogenesis of epidermal nevus syndrome have been reported, along with the identification of *KRAS* and *HRAS* mutations [5, 6]. So far, only one study has described the deletion of the *PTCH* gene in association with sebaceous nevus [7]. This case report presents a novel case of epidermal nevus syndrome with a missense mutation in *PTCH1* gene and cerebral infarction. To our knowledge, there have been no reports of epidermal nevus syndrome with mutation of *PTCH1* gene and cerebral infarction.

## Case presentation

**Fig. 1:**
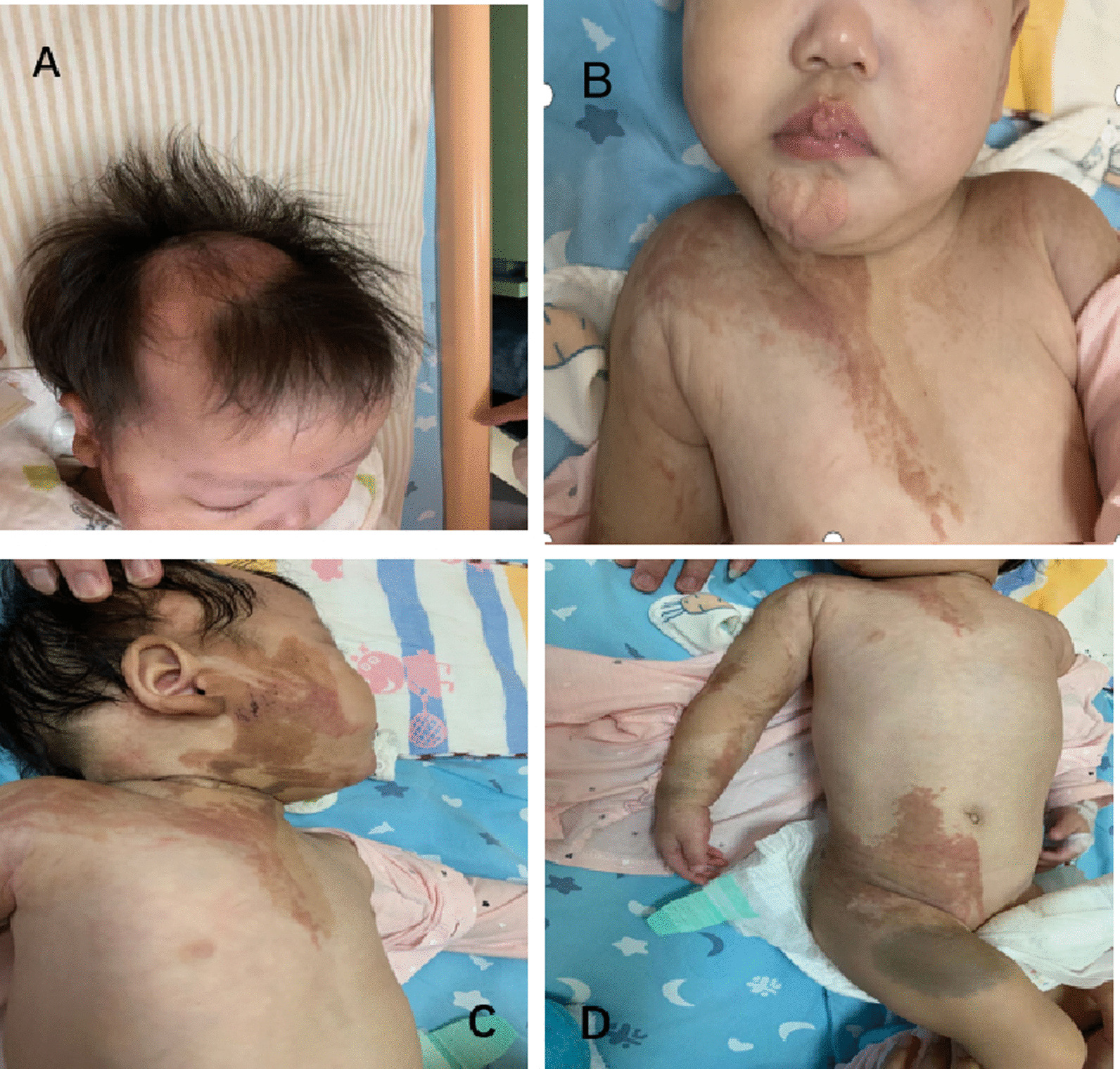
Cutaneous manifestations of patient. **A** Flat, hairless skin lesion on the scalp; **B** anomalies of eye and lip, skin-colored verrucose tumors on the lip; **C** brownish-black and verrucose plaques on the right side of face and cervical nevi; **D** linear skin lesion on right trunk and extremities

**Fig. 2:**
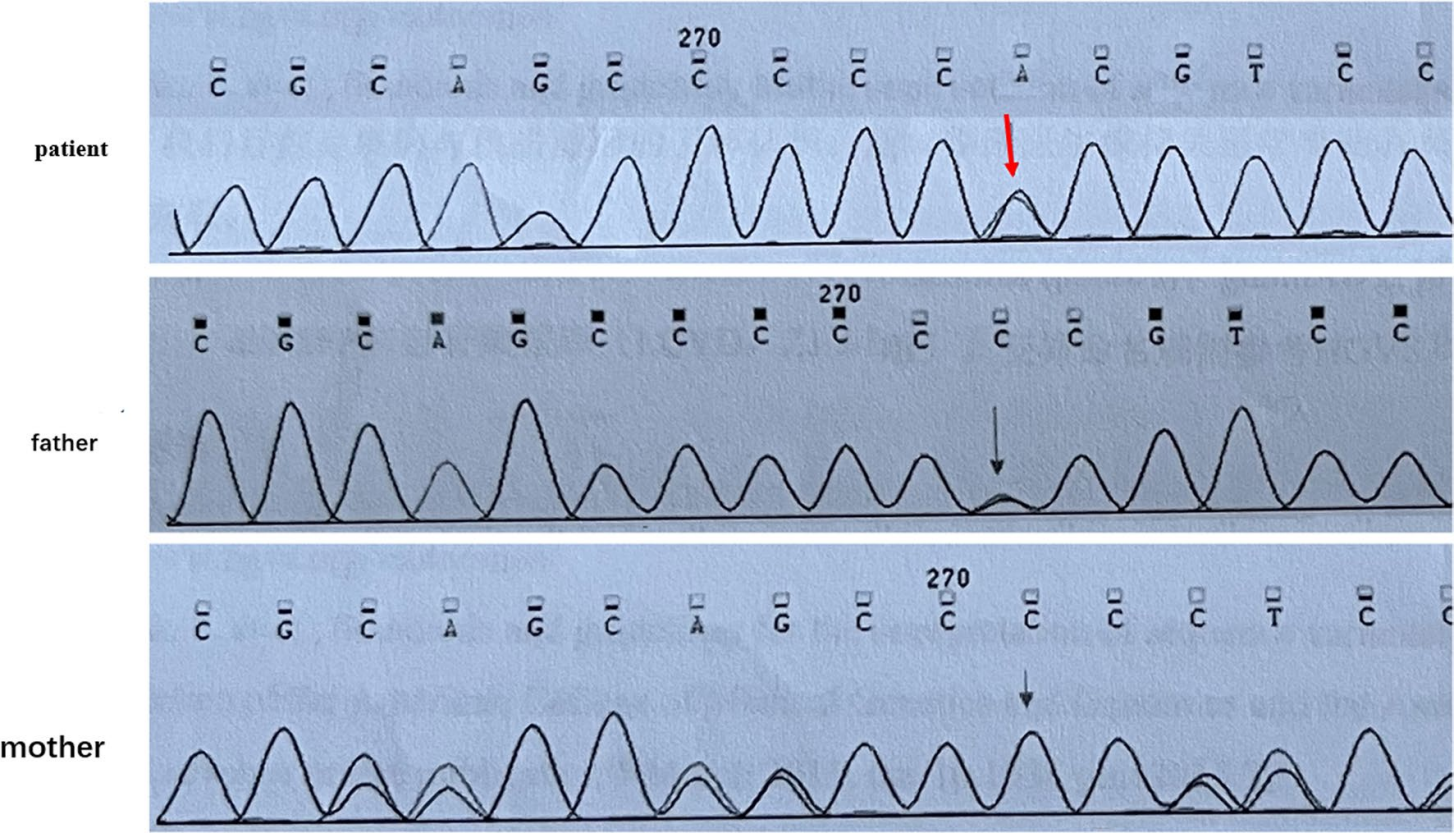
Genetic testing results of the *PTCH1* gene for the patient and his parents. The arrow indicates the position of the deleted nucleotide “C” in the patient. *PTCH1/NM-000264.3* missense mutation, chr9:98270535. c.109G > T variant, *de novo*, which changes Trp to Gly in codon

We present the case of a 10-month-old Chinese female delivered at a gestational age of 35 weeks following an uneventful pregnancy with parents unrelated to each other. No family history of congenital diseases was found. At birth, she presented with a flat, hairless, yellow-brown linear skin lesion in the right central and temporal scalp, accompanied by brownish-black and verrucose plaques on the right hemifacial and cervical nevi together with the right conjunctival lipomatosus. A skin-colored verrucose tumor was found on the lip, as well as extensive brownish-black plaques and brown nevi on the right side of the trunk and the right arm (Fig. 1). Histopathological examination of the plaque lesions on the hip revealed hyperkeratosis, acanthosis, and sebaceous gland hyperplasia. Renal Doppler ultrasound revealed right kidney enlargement with polycystic kidney. Electrocardiogram suggested premature atrial syndrome. Brain magnetic resonance imaging (MRI) was normal at 3 months of age. Genetic testing was performed in her first month, detecting no pathogenic genes related to the clinical symptoms but one clinically significant variant (*PTCH1/NM-000264.3* missense mutation, chr9: 98270535, father source) (Fig. 2).

**Fig. 3:**
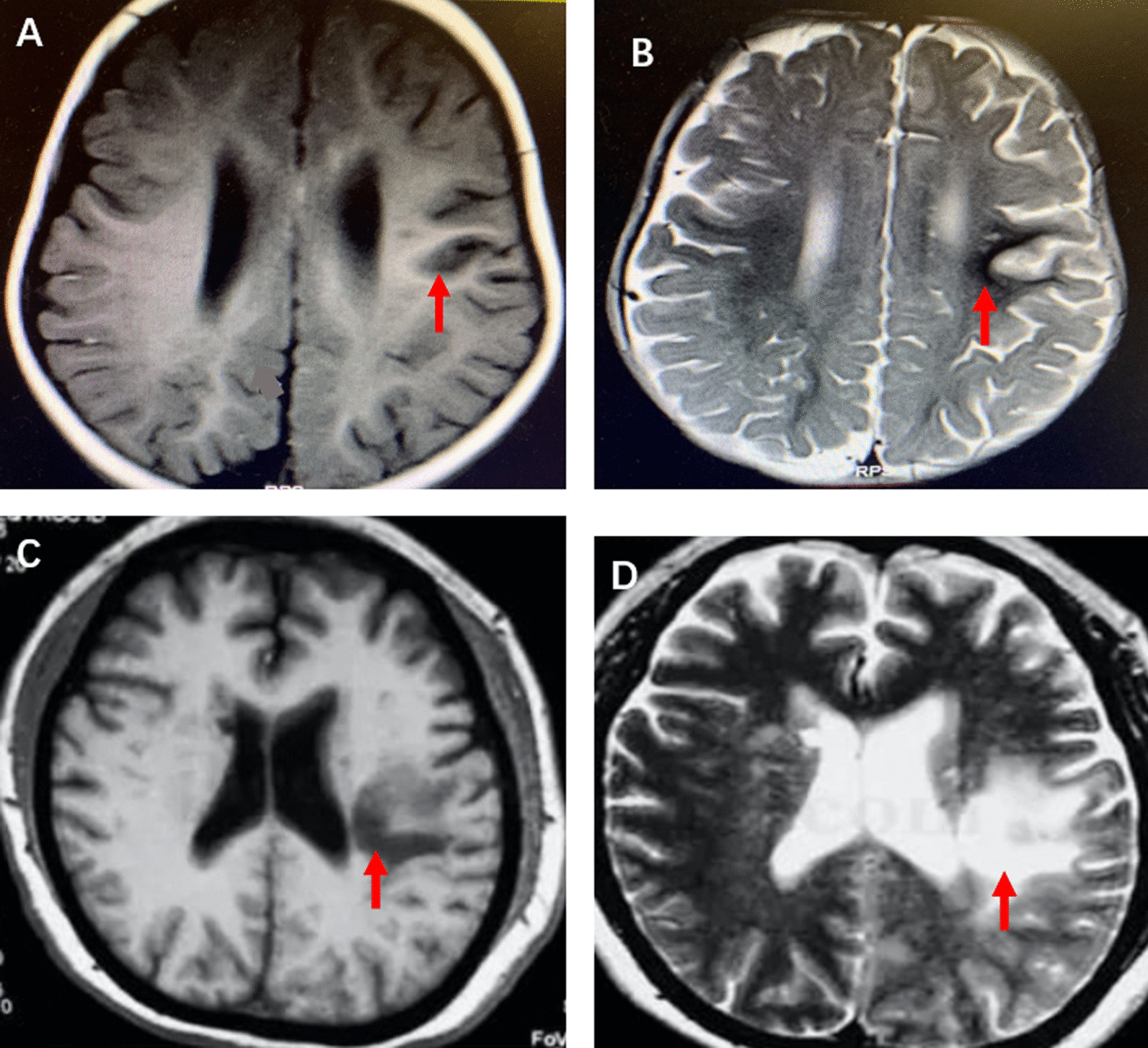
Cranial MRI showing cerebral infarction. The areas of restricted diffusion compatible with hypointense infarction in the left temporal area (**A**) and hyperintense infarction on apparent diffusion coefficient mapping (**B**). New large cerebral infarction in the left hemisphere [T1-WI (**C**) and T2-WI (**D**)]. Arrows show the areas of cerebral infarction

Ten months later, the patient was referred to our department because of mobility disorder in the right limbs and recurrent seizures. Epilepsy started at 10 months of age and presented with medical refractory seizures, accompanied by quadriplegia, mental retardation, and difficulty swallowing. Blood pressure, electroencephalogram (EEG) recordings, and radiographs of long bones were normal. Cranial computed tomography (CT) scan performed owing to the mobility disorder of the right limbs showed widened sulci in the right parietal lobe. Brain MRI performed owing to the quadriplegia showed cerebral infarction (Fig. 3). Paralysis was aggravated with frequent uncontrollable convulsions during her hospitalization, Her parents gave up therapy on day 11, and the patient was discharged home.

Follow-up was carried out by telephone every 3 months, which involved inquiry about limb motor ability and seizures. Currently, after 9 months of follow-up, the patient is still alive but with severe paralysis, mental retardation, and seizures.

## Discussion and conclusions

Our patient not only showed the classical pigmented lesions and various developmental abnormalities of multiple organs, but also demonstrated a missense mutation in *PTCH1* gene and cerebral infarction. Among the current published case reports of ESN, this case is novel, manifested by epidermal nevus syndrome with a mutation of *PTCH1* gene and cerebral infarction.

In accordance with the majority of patients with ENS, the pigmented lesions in our patient are located on the face, neck, truncus, and extremity cutaneous lesions [8], accompanied by various developmental abnormalities of the skin, eyes, and nervous, cardiac, and urogenital systems [9, 10]. Our patient displayed severe neurological clinical manifestations including medical refractory seizures, mental retardation, and severe quadriplegia, indicative of epidermal nevus syndrome [11]. Meanwhile the missense mutation c.109G > T in *PTCH1* gene was tested at birth, and cerebral infarction was discovered at 10 months.

The etiology and pathogenesis of epidermal nevus syndrome spectrum is unclear. In terms of the genetic basis of ENS, the published evidence suggests that genomic mosaicism is a basic feature of ENS [12], The genes involved in the pathway include *KRAS*, *HRAS*, *NRAS*, and *FGFR1*. *FGFR2* and *FGFR3* mutations have previously been reported to be involved in some cases [6, 13]. In a previous report, the sebaceous nevus was associated with deletions of the *PTCH* gene [7]. However, *PTCH1* gene analyses were not performed in ENS. We provide the first evidence of the involvement of the gene *PTCH1* in ENS. However, why did her father have the same missense mutation but no clinical manifestations? Is this an effect of modified genes or environmental factors? Whether the *PTCH1* gene is associated with ENS should be addressed in future studies.

Another significant clinical feature of this case was cerebral infarction. Up to now, in literature, several vascular malformations with ENS have been reported: bilateral renal artery stenosis, bilateral vertebral artery occlusion, unilateral renal artery stenosis, coarctation of the aorta, and azygos anterior cerebral artery and internal carotid artery occlusion [1, 14]. Nevertheless, only one case of cerebral infarction has been reported so far [15]. For our patient, brain magnetic resonance angiography (MRA) should have been performed earlier to clarify whether vascular malformation was present. However, her parents refused further inspection and treatment.

In conclusion, we present a novel case report that demonstrates a missense mutation of *PTCH1* gene (c.109G > T) and cerebral infarction in a female infant with ENS. There is no ideal treatment for ENS. ENS should be considered in children born with nevus sebaceous. ENS is associated with a wide range of abnormalities, and careful evaluation of multiorgan and gene tests is necessary. Our case report may contribute to characterizing the phenotype of ENS, create an awareness among clinicians, raise clinical suspicion, and improve early therapy.

## Data Availability

Not applicable.

## Abbreviations

ENS: Epidermal nevus syndrome
MRI: Magnetic resonance imaging
EEG: Electroencephalogram
MRA: Magnetic resonance angiography
